# Stress Recovery Effects of High- and Low-Frequency Amplified Music on Heart Rate Variability

**DOI:** 10.1155/2016/5965894

**Published:** 2016-08-30

**Authors:** Yoshie Nakajima, Naofumi Tanaka, Tatsuya Mima, Shin-Ichi Izumi

**Affiliations:** ^1^Department of Nursing, School of Health Sciences, Tokai University, Kanagawa, Japan; ^2^Department of Physical Medicine and Rehabilitation, Tohoku University Graduate School of Medicine, Sendai, Japan; ^3^Department of Rehabilitation, Teikyo University Chiba Medical Center, Chiba, Japan; ^4^Graduate School of Core Ethics and Frontier Sciences, Ritsumeikan University, Kyoto, Japan; ^5^Department of Physical Medicine and Rehabilitation, Tohoku University Graduate School of Biomedical Engineering, Sendai, Japan

## Abstract

Sounds can induce autonomic responses in listeners. However, the modulatory effect of specific frequency components of music is not fully understood. Here, we examined the role of the frequency component of music on autonomic responses. Specifically, we presented music that had been amplified in the high- or low-frequency domains. Twelve healthy women listened to white noise, a stress-inducing noise, and then one of three versions of a piece of music: original, low-, or high-frequency amplified. To measure autonomic response, we calculated the high-frequency normalized unit (HFnu), low-frequency normalized unit, and the LF/HF ratio from the heart rate using electrocardiography. We defined the stress recovery ratio as the value obtained after participants listened to music following scratching noise, normalized by the value obtained after participants listened to white noise after the stress noise, in terms of the HFnu, low-frequency normalized unit, LF/HF ratio, and heart rate. Results indicated that high-frequency amplified music had the highest HFnu of the three versions. The stress recovery ratio of HFnu under the high-frequency amplified stimulus was significantly larger than that under the low-frequency stimulus. Our results suggest that the high-frequency component of music plays a greater role in stress relief than low-frequency components.

## 1. Introduction

Cerebrovascular disease (CVD) is a serious global health problem. Unfortunately, in many patients, recovery is limited by various degrees of sequelae. In addition to physical handicaps, stroke survivors often face pain, anxiety, and mental strain. These challenges can affect motivating, slowing, or preventing rehabilitation during both the acute and chronic phases. For the management of CVD in terms of social integration, it can be beneficial to teach patients psychological stress-coping strategies. For this purpose, various relaxation techniques have been proposed.

Because many CVD patients receiving rehabilitation suffer from cognitive and emotional problems in addition to impaired physical functioning [1], relaxation techniques, which do not require patient training or active effort, can be very useful. Music therapy enhances the state of psychological and physiological relaxation through passive listening [2] and is thought to contribute to the mental and physical wellbeing of patients. For example, calm music can alleviate stress [3], and incorporating music into rehabilitation programs can improve depression, mobility, and cognitive function [4]. However, owing to individual variations in music preference, even in the same genre, it has been difficult to examine the precise effects of music on mental status [5].

The individual relaxation response to music is affected by music genre, which is composed of different basic compositional elements, such as melody, rhythm, harmony, and tonality. Previous research has shown relaxation effects of classical music at both the subjective and objective levels [6]. Additionally, studies comparing classical baroque and heavy metal music have indicated that the different rhythms have different effects on autonomic nervous system (ANS) function [7, 8]. Classical baroque music has also been found to decrease activity in the sympathetic nervous system (SNS) [9]. However, the precise effects of specific compositional elements of music on the ANS and relaxation responses have not been clarified.

Although a direct comparison is not possible owing to differences in the audible frequency range in rats (0.1–70,000 Hz) and humans (20–20,000 Hz), a study with rats showed that SNS activity was suppressed when rats were exposed to music in which the 4–16 kHz frequency band was amplified [10]. Thus, the different frequency characteristics of music may play a modulatory role in human ANS function.

To evaluate ANS activity in the present study, we assessed heart rate variability (HRV) as a differential measure of SNS [11] and parasympathetic nervous system (PNS) activity [12] with good temporal resolution.

We sought to clarify the relaxing effects of pieces of music with different frequency contents via measurement of ANS activity. We hypothesized that this parameter of music would affect human relaxation by modifying PNS activity. We quantitatively compared various indices of ANS for 3 types of short music clips with amplification of high-frequency components (HFM) and low-frequency components (LFM) and without processing (original music: OM). We presented a brief uncomfortable sound (scratching sound) stimulus as a stressor.

## 2. Participants and Methods

### 2.1. Participants

Twelve healthy right-handed female university students, aged 21-22 years, volunteered for this study. Exclusion criteria included a history of central nervous system, psychiatric or endocrine disorders, current treatment with medication, and a history of smoking or professional musical training, according to self-report. All participants had experience playing the piano as a hobby for an average of 9.4 ± 3.8 years. On the night preceding the study, participants were asked to avoid alcohol and get adequate sleep, and, on the day of the study, they were asked not to ingest any caffeine. As estrogen and progesterone can both affect ANS activity, the study day was scheduled such that it did not coincide with menstrual or ovulation periods.

All participants consented to participate in the present study, which was approved by the Ethical Committee of the relevant institution according to the Declaration of Helsinki. Participants provided written informed consent after receiving full oral and written explanations about the purpose and methods of the study.

### 2.2. Experimental Procedure

The experiment was performed at a room temperature of 24°C. Environmental noise was measured with a sound meter (LA-5560, Ono Sokki Co., Ltd., Kanagawa, Japan) and adjusted so that it remained below 50 dB. Participants sat on a reclining chair. We measured blood pressure and body temperature after 5 minutes of rest. We then initiated electrocardiogram measurements.

**Figure 1:**
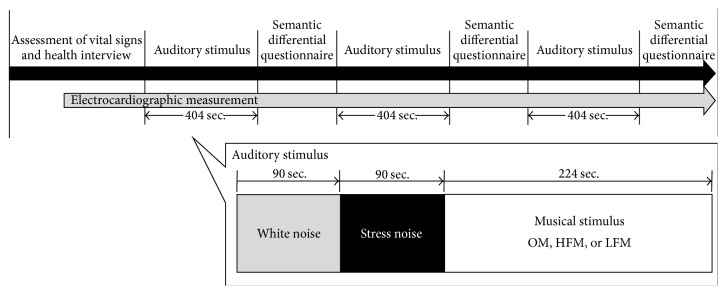
Experimental procedure. One of three musical stimuli (OM, HFM, and LFM) was selected in a counterbalanced random order. The participants were asked to complete the semantic differential questionnaire after listening to each piece of music. HFM: music with an amplified high-frequency component; LFM: music with an amplified low-frequency component; OM: original music.

Figure 1 shows a schema of the experimental paradigm. Participants listened to 90 seconds of white noise (WN) and 90 seconds of a stress-inducing friction sound, generated by scratching a blackboard (stressful noise: SN). They then listened to the musical stimulation (MS). Auditory stimuli (AS) were presented using a portable recorder (R-09, Roland Corporation, Shizuoka, Japan) and headphones (MDR-CD900ST, Sony Corporation, Tokyo, Japan). The frequency of the reproduction equipment used in this study ranged from 20 Hz to 22 kHz for the portable recorder and from 5 Hz to 30 kHz for the headphones. The sound pressure level was between 50 and 70 dB.

Three types of MS (OM, HFM, and LFM) were presented to each participant in a random order. After listening to the MS, participants used the semantic differential (SD) method to evaluate impressions.

### 2.3. Music Stimuli

The piece chosen for the musical task was the third movement of Horn Concerto No. 2, in E-flat major (composed by Wolfgang Amadeus Mozart, K.417). The piece was 224 seconds long, with a speed of about 98 bpm. It was played at a fixed tempo without rubato, that is, speeding up or slowing down. We confirmed that this concerto was unfamiliar to the participants.

For HFM, frequency bands 3.5 kHz or greater were amplified by approximately 6 dB (2-fold). For LFM, we did not process the central frequency band from 0.5 Hz to 0.8 kHz, so as to maintain the impression of the OM. Instead, we amplified the low and medium registers below 0.5 Hz by approximately 12 dB (4-fold). HFM and LFM were processed in accordance with the hearing characteristics of humans [13, 14].

Music software (Singer Song Writer 8.0 VS for Windows, Internet Co., Ltd., Osaka, Japan) was used to process the frequencies of the music clip. This software has a processing frequency range of 0.25–16 kHz and a processing sound pressure amplification capability of 0.25–4 times, in the range of 12 dB in the specified frequency bands.

### 2.4. Physiological and Psychological Evaluation

To evaluate ANS activity, we analyzed HRV. We conducted frequency analyses of electrocardiogram waveforms using a heart rate monitor (LRR-03, GMS Co., Ltd., Tokyo, Japan). We used MemCalc analysis system (Tarawa, GMS Co., Ltd.) for real-time spectral analysis with the maximum entropy method. This software calculates heart rate (HR) spectral power from pulse trains composed of the R-R intervals of four heartbeats. The software can separately analyze very low-frequency (LF) components (below 0.04 Hz), LF components (0.04–0.15 Hz), and high-frequency (HF) components (0.15–0.40 Hz). The sampling frequency was set at 250 Hz.

Because HF components also respond to activity in the respiratory center, caution is required when measuring HRV [15]. To control for respiratory condition, we discontinued the experiment if a participant had a maximum respiratory interval shorter than 7 seconds, respiratory frequency below nine breaths/min, or breathing rate greater than 24 breaths/min.

In this study, we analyzed the following indices of HRV. HR is an index of both SNS activity and PNS activity. Increased HR indicates increased SNS activity, while decreased HR indicates increased PNS activity. For PNS activity, we used normalized value HFnu, calculated by dividing the sum of HF and LF by HF. We used the LF/HF ratio as an index for SNS activity. The sum total of all autonomic nervous system activity was denoted by the normalized value LFnu, calculated by dividing the sum of HF and LF by LF. The stress recovery ratio, which was a normalized index of stress recovery, was defined as the ratio of change for each HRV index. This was calculated by subtracting SN from WN and dividing the HRV index observation values by the value obtained by subtracting SN from MS.

In addition to the HRV measurements, subjective evaluations of the emotional impact of the music stimuli were determined using a modified SD method [16, 17]. Fourteen pairs of adjectives were assessed on seven-point scales, including “loud–soft,” “beautiful–ugly,” “pure–impure,” “hard–soft,” “sharp–dull,” “strong–weak,” “deep–metallic,” “annoying–not annoying,” “mild–gruff,” “pleasant–unpleasant,” “powerful–unsatisfactory,” “pleasing–unpleasing,” “shrill–calm,” and “noisy–quiet” [18, 19]. The SD rating test was performed after listening to MS.

### 2.5. Statistical Analysis

We used a generalized estimating equation (GEE) approach for the HRV indices of HFnu, LFnu, the LF/HF ratio, and HR during each MS. The model included AS (WN, SN, and MS), the MS (OM, HFM, and LFM), and the presentation order as an intrasubject variable. We used predictive factors to analyze the main effects and the interaction between AS and MS [20]. The durations of WN, SN, and MS were used as scale-weighting functions, and we performed replicate measurement in which we exchanged the stimulus correlation matrix.

After GEE analysis, we further investigated the differences in the modification of stress recovery produced by frequency modulated music clips. Specifically, we used stress recovery ratios, that is, the ratio of the HRV parameters during MS and WN conditions, with that during the SN condition as a baseline:

(1) $$\begin{array}{c} \text{Stress recovery ratio}=\frac{\left(X\left(MS\right)-X\left(SN\right)\right)}{\left(X\left(WN\right)-X\left(SN\right)\right)}. \end{array}$$

After testing the normality of the data distribution using the Shapiro–Wilk test, we performed nonparametric statistical analysis of the stress recovery ratio values and psychological impressions using the Friedman and the Wilcoxon tests, respectively. This analysis was based on an* a priori* hypothesis that modifying the frequency contents of the music clips would affect the physiological stress response recovery rate following exposure to the uncomfortable sound.

Psychological impressions of the three MS (OM, HFM, and LFM), evaluated using the SD method, were statistically analyzed using the Friedman test.

Statistical analysis was performed using the software SPSS ver. 19 (IBM Corporation) and the level of significance for each test was set at 0.05.

## 3. Results

A summary of the GEE results for the HRV parameters is shown in Table 1. For the main effect of AS, we observed significant differences for the HFnu, LFnu, and LF/HF ratio (*p* < 0.001) but not for HR (*p* = 0.126). For the main effect of MS, we found a significant difference for HFnu only (*p* < 0.001). For the main effect of MS presentation order, no significant differences were observed. The interaction between AS and MS was significant only for HR (*p* = 0.039).

**Figure 2:**
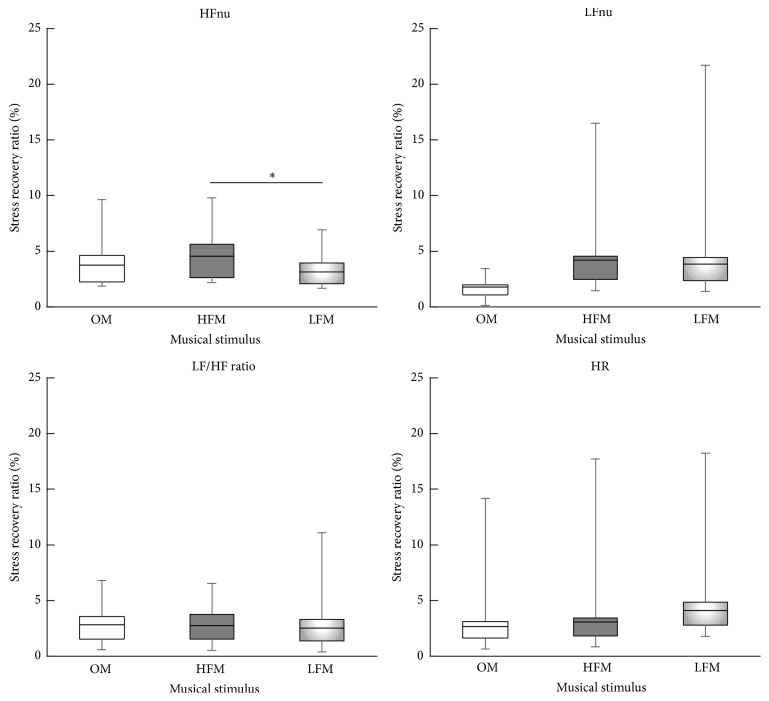
Stress recovery ratio of heart rate variability index. Box plot showing median (central lines) and 25% and 75% quartile ranges around the median (box width) and upper and lower limits. Error bars indicate standard deviation. ^*∗*^ *p* < 0.05; HFM: music with an amplified high-frequency component; HFnu: high-frequency normalized unit; HR: heart rate; LF/HF: low-/high-frequency ratio; LFM: music with an amplified low-frequency component; LFnu: low-frequency normalized unit; OM: original music.

To further clarify the role of frequency components in relaxation, we analyzed stress recovery ratios for HFnu, LFnu, LF/HF ratio, and HR. The stress recovery ratios are summarized in Figure 2. The stress recovery ratio of HFnu for HFM was significantly larger than that for LFM (*p* = 0.049). However, the differences between those for HFM and OM and those for LFM and OM were not significantly different. We found no significant differences in the ratios of the other HRV indices among the three MS tasks.

Respiratory rates were considered to reflect the respiratory conditions during the experiment.

Psychological impressions of MS were not significantly different for any of the 14 items (Table 2).

## 4. Discussion

We found that the stress recovery ratio, as reflected by the PNS activity, was significantly higher after listening to HFM compared with LFM or OM. This result indicated that enhancing the high-frequency components of music might be useful in alleviating stress. Although previous studies have clarified the stress recovery effects of music [21, 22], no reports have demonstrated that the frequency properties of music are relevant to the modification of stress-related physiological changes [23]. One rat study [10] investigated physiological changes induced by high-frequency sounds via dopamine-synthesis. Supporting our observations in humans, the researchers found that only music with amplified sounds in the high-frequency band could modulate ANS activity and decrease blood pressure. Accordingly, artificially increasing the high-frequency sounds in music might be an efficient and economical way to modulate ANS activity, leading to stress recovery. We used a short music stimulus composed by Mozart, whose pieces of music are rich in high-frequency components [24]. However, it is necessary to be cautious about directly comparing rats with humans due to species-specific differences in hearing capacity [25, 26]. That we found a modulation of the stress recovery effect for HFM only indicates that the enhancement of high-frequency components might have induced special biological effects. However, the precise physiological mechanisms of HFM on ANS activity are beyond the scope of the present study.

In humans, auditory sensitivity for high-frequency sounds around 4 kHz is better than that for other frequency bands [13, 14]. Although older adult listeners often have high-frequency hearing loss [27], the participants in the present study were young and had no problems in terms of sensitivity to the amplified frequency band. In addition, the fundamental frequencies of the three music stimuli were not significantly different, as confirmed by the lack of significant differences in music impressions. Although we hypothesized that the HFM would change the stress recovery via modulation of PNS activity, there are other possibilities, such as those related to cognitive or psychological factors associated with music perception [28] or linguistic and musical syntactic processing [29].

Regarding the parameters of the ANS responses calculated from HRV, LF components correspond to Mayer waves, observed in the 0.1 Hz cycle of systolic blood pressure. Mayer waves mediate baroreceptors in the carotid sinus, affecting the efferent pathway of the cardiac vagal or sympathetic nerves to inhibit rhythm in the sinus node. Accordingly, LF components reflect both the cardiac vagal system and cardiovascular SNS activity [30]. For LFnu, we found a significant effect of AS, suggesting that the stress response was induced by the stress sound stimulation and its recovery was associated with the MS. However, it is not possible to individually separate SNS from PNS function in terms of the LFnu results, because this variable reflects activity in both systems.

Regarding HR, we found no significant effects of AS, suggesting that the HR is not sufficiently sensitive to detect the stress-related HRV responses associated with an uncomfortable SN.

The LF/HF ratio, which reflects SNS activity, increased with the stress stimulation and decreased with the MS, as expected. The SNS activity response plateaus approximately 90 seconds after exposure to an auditory stress stimulus [31], and the nervous response brought about by music recognition appears after listening to music for approximately 150 seconds [32]. Because the MS used in this study was 224 seconds long, we expected that the time window of the present experimental paradigm was suitable for the SNS response. Additionally, the effects of stress recovery modification in the SNS, if any, were likely minimal. Because HRV is a more sensitive reflection of PNS compared with SNS changes [33], HRV may not have reflected changes in the LF/HF ratio, which is an index of SNS activity. Accordingly, in future research, it will be necessary to evaluate other indices of SNS activity, such as electrodermal response [34].

Complex compound sound information, containing high-frequency components and music, is transmitted to the primary auditory cortex through the inferior colliculus [35]. However, the exact anatomical mechanisms by which ANS activity is modulated have not been clarified. Further studies with animal models or human neuroimaging might be necessary to address this issue.

This study had a number of limitations. The first is that we only presented one piece of music as a MS. Indeed, different HFM stress recovery effects may be observed with different songs. Because Mozart is very popular in Japan, we expect that the music clip used in the present study can be regarded as a typical example. However, we cannot exclude the possibility that other music clips, for example, those of Japanese music, might have different effects on relaxation. Moreover, because our participants were healthy, young individuals, the results cannot directly be applied to elderly individuals, who often have markedly decreased hearing ability for high frequencies, that is, 4 kHz–8 kHz [36]. In the present study, we recruited only participants with experience playing piano. Thus, our results might be different if we examined individuals with no experience playing music. Furthermore, patients with CVD might react differently to music stimuli. For instance, CVD patients with depression might behave differently because their baseline psychological states are different from those of healthy volunteers. In addition, it would be difficult to assess autonomic function from ECG in CVD patients with autonomic dysfunction, such as those with severe diabetic neuropathy. These points should be addressed with respect to future clinical applications.

Previous studies have reported large individual variations in HRV analysis [37]. Accordingly, we failed to observe an interaction between AS and MS. One possible solution may be the use of a nonmusic control for MS as a baseline stimulus. However, the subjective impression elicited by MS and a nonmusic stimulus would be different, making simple and direct comparisons between MS and nonmusic controls difficult.

Our planned comparison, based on an* a priori* hypothesis, revealed significant effects of frequency modulation of music clips on the stress recovery rate of HFnu. However, although the stress recovery ratio for HFM was significantly larger than that for LFM, this difference was only a trend compared with the stress recovery ratio associated with OM. This divergence might be due to the small sample size and large inter-individual variability. It should be noted that the stress recovery ratio of HFnu for LFM was the smallest of the three conditions, potentially reflecting the negative effects of low-frequency components below 0.5 kHz [38]. Further research including a larger sample (more than 12 participants) would be helpful in addressing these limitations.

An increasing number of studies have focused on the biological effects of nonstationary high-frequency components, including those above the human audible range [39, 40]. Such sounds may be processed in the midbrain and diencephalon. Because these “hypersonic effects” are clearly observed in gamelan music, further research comparing Western and gamelan music might be fruitful [41].

Changing the frequency contents of music to enhance the stress recovery effects could have useful clinical applications. For example, noise exposure increases the risk of coronary heart disease [42], while listening to music decreases the rate of CVD [43]. Furthermore, to increase the training effects of rehabilitation, music could be used to induce relaxation before and after therapy. As a means of stress management that patients could perform themselves, HF-amplified preferred music could be generated and provided to patients, who could listen to it when feeling stressed. In conclusion, listening to HF-amplified music increased PNS activity, measured via the stress recovery ratio more than LF-amplified music. These findings expose possibilities for various clinical applications.

## Figures and Tables

**Table 1:** Heart rate variability analysis.

	Auditory stimulus	Musical stimulus	Heart rate variability index (mean ± SD)			
			HFnu (ratio)	LFnu (ratio)	LF/HF (ratio)	HR (bpm/min)
	WN	OM	0.40 ± 0.13	0.67 ± 0.11	2.01 ± 1.72	77.95 ± 8.99
		HFM	0.42 ± 0.12	0.60 ± 0.16	1.87 ± 1.41	78.51 ± 8.47
		LFM	0.47 ± 0.16	0.63 ± 0.18	1.91 ± 2.23	77.03 ± 9.12

	SN	OM	0.25 ± 0.11	0.50 ± 0.19	4.38 ± 3.27	79.17 ± 8.05
		HFM	0.27 ± 0.10	0.58 ± 0.15	4.67 ± 3.79	78.91 ± 9.27
		LFM	0.30 ± 0.13	0.58 ± 0.18	4.30 ± 2.80	79.62 ± 8.28

	MS	OM	0.46 ± 0.13	0.55 ± 0.15	1.65 ± 1.11	76.80 ± 9.34
		HFM	0.52 ± 0.14	0.53 ± 0.14	1.48 ± 1.66	78.61 ± 9.80
		LFM	0.51 ± 0.11	0.52 ± 0.16	1.33 ± 0.95	75.46 ± 8.97

GEE *p* value	Auditory stimulus		0.001^*∗*^	0.001^*∗*^	0.001^*∗*^	0.13
	Music stimulus		0.001^*∗*^	0.98	0.74	0.13
	Auditory stimulus × music stimulus		0.68	0.07	0.98	0.04^*∗*^
	Order effect		0.69	0.37	0.22	0.96

^*∗*^ *p* < 0.05; HFM: music with an amplified high-frequency component; HFnu: high-frequency normalized unit; HR: heart rate; LF/HF: low-/high-frequency ratio; LFM: music with an amplified low-frequency component; LFnu: low-frequency normalized unit; MS: musical stimulus; OM: original music; SD: standard deviation; SN: stress noise.

**Table 2:** Semantic differential scales.

	Semantic differential scales (mean ± SD)			Friedman test
	OM	HFM	LFM	*p* value
Loud–soft	4.23 ± 0.76	4.46 ± 0.72	4.23 ± 0.5	0.368
Beautiful–ugly	4.69 ± 0.95	5.08 ± 1.03	4.85 ± 0.95	0.301
Pure–impure	4.92 ± 0.49	4.69 ± 1.14	5.00 ± 0.37	0.629
Hard–soft	3.31 ± 1.09	3.85 ± 0.69	3.15 ± 0.95	0.097
Sharp–dull	4.15 ± 0.49	4.46 ± 0.86	3.85 ± 0.72	0.179
Strong–weak	4.85 ± 0.43	5.23 ± 0.69	4.62 ± 0.87	0.061
Deep–metallic	5.00 ± 1.21	4.77 ± 1.26	5.00 ± 0.9	0.717
Annoying–not annoying	3.08 ± 1.49	3.77 ± 1.5	3.15 ± 1.01	0.072
Mild–gruff	5.23 ± 1.26	4.77 ± 1.32	5.31 ± 1.29	0.261
Pleasant–unpleasant	5.85 ± 1.04	5.31 ± 1.66	6.00 ± 0.62	0.174
Powerful–unsatisfactory	5.08 ± 0.95	5.62 ± 1.21	5.46 ± 1.22	0.368
Pleasing–unpleasing	5.92 ± 1.19	5.54 ± 1.41	5.92 ± 0.95	0.593
Shrill–calm	4.23 ± 1.38	4.31 ± 1.26	3.77 ± 1.08	0.227
Noisy–quiet	4.46 ± 0.85	4.69 ± 0.95	4.38 ± 0.64	0.229

HFM: music with an amplified high-frequency component; LFM: music with an amplified low-frequency component; OM: original music; SD: standard deviation.
